# Comparative analysis of long noncoding RNAs in angiosperms and characterization of long noncoding RNAs in response to heat stress in Chinese cabbage

**DOI:** 10.1038/s41438-021-00484-4

**Published:** 2021-03-01

**Authors:** Xiaoming Song, Jingjing Hu, Tong Wu, Qihang Yang, Xuehuan Feng, Hao Lin, Shuyan Feng, Chunlin Cui, Ying Yu, Rong Zhou, Ke Gong, Tong Yu, Qiaoying Pei, Nan Li

**Affiliations:** 1 College of Life Sciences/Center for Genomics and Bio-computing, North China University of Science and Technology, Tangshan, Hebei China; 2 Food Science and Technology Department, University of Nebraska-Lincoln, Lincoln, NE USA; 3 School of Life Science and Technology and Center for Informational Biology, University of Electronic Science and Technology of China, Chengdu, China; 4 Department of Food Science, Aarhus University, Aarhus, Denmark

**Keywords:** Heat, RNA sequencing, Gene expression, Non-coding RNAs

## Abstract

Long noncoding RNAs (lncRNAs) are widely present in different species and play critical roles in response to abiotic stresses. However, the functions of lncRNAs in Chinese cabbage under heat stress remain unknown. Here, we first conducted a global comparative analysis of 247,242 lncRNAs among 37 species. The results indicated that lncRNAs were poorly conserved among different species, and only 960 lncRNAs were homologous to 524 miRNA precursors. We then carried out lncRNA sequencing for a genome-wide analysis of lncRNAs and their target genes in Chinese cabbage at different stages of heat treatment. In total, 18,253 lncRNAs were identified, of which 1229 differentially expressed (DE) lncRNAs were characterized as being heat-responsive. The ceRNA network revealed that 38 lncRNAs, 16 miRNAs, and 167 mRNAs were involved in the heat response in Chinese cabbage. Combined analysis of the *cis*- and *trans*-regulated genes indicated that the targets of DE lncRNAs were significantly enriched in the “protein processing in endoplasmic reticulum” and “plant hormone signal transduction” pathways. Furthermore, the majority of *HSP* and *PYL* genes involved in these two pathways exhibited similar expression patterns and responded to heat stress rapidly. Based on the networks of DE lncRNA-mRNAs, 29 and 22 lncRNAs were found to interact with *HSP* and *PYL* genes, respectively. Finally, the expression of several critical lncRNAs and their targets was verified by qRT-PCR. Overall, we conducted a comparative analysis of lncRNAs among 37 species and performed a comprehensive analysis of lncRNAs in Chinese cabbage. Our findings expand the knowledge of lncRNAs involved in the heat stress response in Chinese cabbage, and the identified lncRNAs provide an abundance of resources for future comparative and functional studies.

## Introduction

The central dogma of molecular biology indicates that RNA acts as a messenger molecule to transfer genetic information from DNA to proteins^1^. However, >75% of transcripts in higher eukaryotic genomes are not translated into proteins and are classified as noncoding sequences^2,3^. Among them, the group of RNA transcripts whose length is longer than 200 nt are defined as long noncoding RNAs (lncRNAs)^4^. Compared with that of mRNAs, the abundance of lncRNAs is low and has strong tissue and cell expression specificity^4^. They regulate gene expression at the transcriptional, posttranscriptional, epigenetic, and other levels^5–7^.

With the development of sequencing technology, a growing number of lncRNAs have been revealed in several plant species, such as *Arabidopsis thaliana*^8,9^, *Oryza sativa*^10,11^, *Zea mays*^12,13^, *Solanum lycopersicum*^14,15^, and *Medicago truncatula*^16^. In addition, some databases have been developed to store plant lncRNAs^17–20^. However, it remains challenging to understand which of the lncRNAs are functional and how their functions are exerted. Comparative analysis of genes across various species would be a powerful tool for studying their functions and modes of action. The degree of conservation is recognized as a key issue in assessing the impact of lncRNAs. It was reported that the sequences of only 6.7% of tomato lncRNAs were conserved with those of potato lncRNAs^21^. Less than two percent of all lncRNAs in Arabidopsis are conserved throughout the plant kingdom^22^. Therefore, it is worth exploring whether lncRNAs are also poorly conserved in additional species.

In plants, the majority of lncRNAs are produced by RNA polymerase II, while the others are transcribed by RNA polymerase III or IV/V^23,24^. Only stable lncRNAs transcribed by RNA polymerase II are considered “typical lncRNAs”^25^. According to their location with respect to the nearest protein-coding genes in the genome, typical lncRNAs can be classified as long intergenic noncoding RNAs (lincRNAs), long noncoding nature antisense transcripts (lncNATs), or long intronic noncoding RNAs (incRNAs)^26^. The protein-coding genes mediated by lncRNAs can be divided into *cis-* and *trans-*models according to the action distance^27,28^. In addition, some lncRNAs interact with microRNAs (miRNAs), serving as miRNA precursors or competing endogenous RNAs (ceRNAs) to serve as decoys for specific miRNAs, thus protecting the target mRNAs from repression^29–32^. In recent years, several studies have indicated that lncRNAs play important roles in various biological processes in plants, including roles in organ development, nutrient metabolism, male sterility, and plant immunity^33–37^. In particular, lncRNAs are considered essential regulators of the response to biotic and abiotic stresses in plants^30,38–40^. For example, the lncRNA *PsiLncRNA00268512* shows dynamic expression changes under heat stress in *Populus simonii*^41^, and in wheat, *TalnRNA27* and *TalnRNA5* exhibit upregulated expression under heat stimulation^42^.

Chinese cabbage (*Brassica rapa* ssp. *pekinensis*) is one of the most important leafy vegetable species in China. It has a long history of cultivation and is enjoyed by people worldwide. Chinese cabbage grows best in cold, cool, and humid climates, as high temperature often affects the formation of leaf balls and induces an increase in susceptibility to infectious disease, which leads to severe declines in yield and quality^43^. Therefore, it is of great importance to elucidate the heat resistance mechanism of Chinese cabbage and develop new cultivars resistant to heat stress. *B. rapa* includes three main subspecies: Chinese cabbage, nonheading Chinese cabbage (NHCC, *Brassica rapa* ssp. *chinensis*), and turnip (*Brassica rapa* ssp. *rapa*). By investigating the transcript profiles, researchers identified approximately 625 genes that are differentially expressed (DE) between the heat-sensitive and heat-tolerant varieties of NHCC^44^. A total of 1031 *cis*-natural antisense transcripts (*cis*-NATs) were detected in Chinese cabbage and NHCC, and the small interfering RNAs (siRNAs) derived from 12 of them were reported to be heat responsive^45^. lncRNAs involved in thermotolerance were functionally characterized in only NHCC^38,46^. However, reports on the comprehensive investigation of lncRNAs involved in heat tolerance are still lacking in Chinese cabbage. Therefore, we carried out this study owing to the important biological functions of lncRNAs in regulating plant heat tolerance.

In this study, based on the plant lncRNA sequences stored in public databases, we conducted a comprehensive comparative analysis of lncRNAs among 37 species. Furthermore, we systematically identified lncRNAs related to heat stress in the whole genome of Chinese cabbage by performing lncRNA sequencing, predicted their potential target genes and analyzed their functions. Our study not only provides valuable information on the evolutionary conservation of lncRNAs in plants but also expands the knowledge of lncRNAs involved in heat stress. The lncRNAs identified in this study constitute an abundant resource for future research on noncoding RNAs.

## Results

### Comprehensive comparative analysis of lncRNAs revealed poor conservation among 37 species

**Fig. 1:**
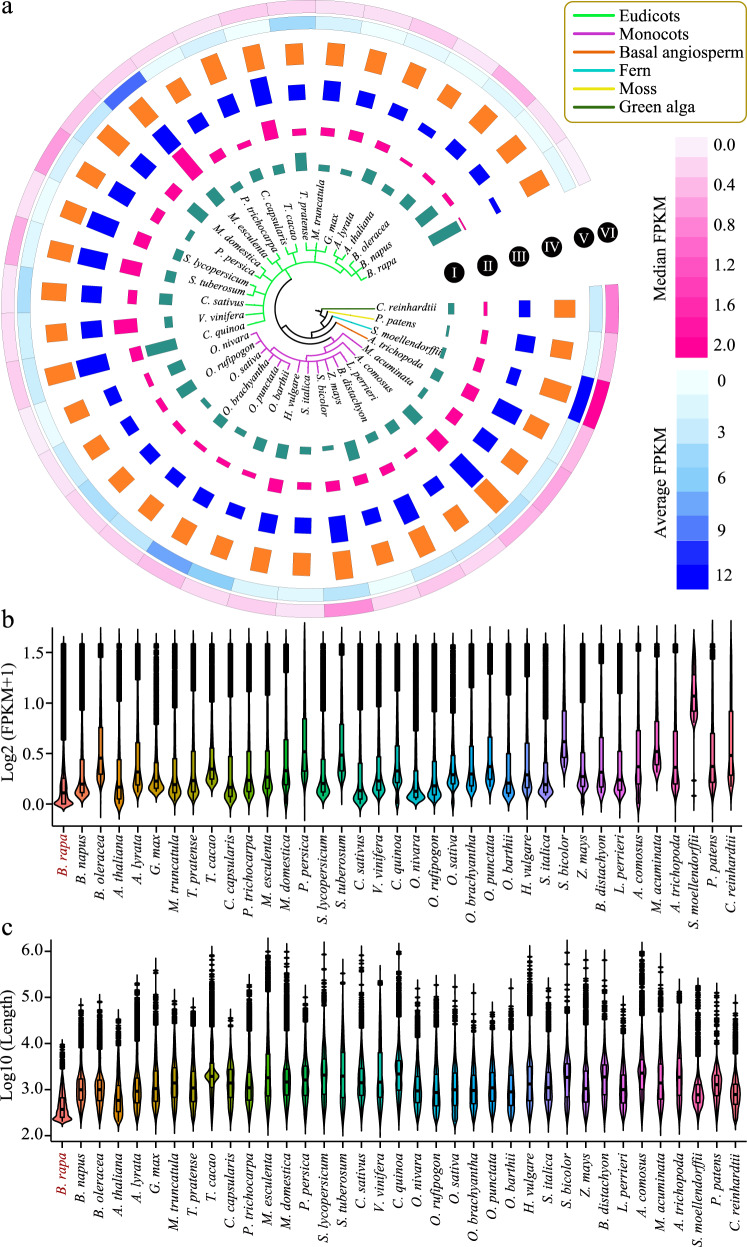
Global comparative analysis of lncRNAs among 37 species. **a** Plot of lncRNA characteristics, including the following: I, the lncRNA number; II, the mean length; III, the median length; IV, the mean exon number; V, the mean expression value; and VI, the median expression value. **b** Violin plots and boxplots of log_2_(FPKM+1) values of lncRNAs with an FPKM<2 in all species. **c** Violin plots and boxplots of the log_10_(length) values of lncRNAs in all species

To comprehensively understand the characteristics of lncRNAs in plants, we collected lncRNAs from 36 species: 18 eudicots, 14 monocots, 1 basal angiosperm, 1 fern, 1 moss, and 1 green alga (Fig. 1). In addition, the lncRNAs of Chinese cabbage were obtained using lncRNA sequencing. Here, a total of 247,242 lncRNAs were detected in all species, with an average number of 6682 (Table S1). Compared with that in other species, the number of lncRNAs detected in Chinese cabbage was the largest (18,253), whereas only 2267 and 1498 lncRNAs were found in fern *Selaginella moellendorffii* and moss *Physcomitrella patens*, respectively. Both the mean length (550.83) and median length (371) of the lncRNAs in Chinese cabbage were less than those of all the other species (Fig. 1, Table S1), and this phenomenon was consistent with previous reports in NHCC^33,38^.

The mean exon number was the lowest (1.83) in Arabidopsis, whereas the highest was found in *Ananas comosus* (3.14). The mean (1.69) and median (0.08) values of expression and median expression in Chinese cabbage were notably lower than the averages in all other species (Fig. 1a, Table S1). Although the expression abundance of most lncRNAs was generally low, we still found that a few lncRNAs had very high expression levels, indicating that specific expression occurred for different lncRNAs (Fig. S1). Moreover, the expression patterns of lncRNAs in many species also significantly differed. For example, the mean FPKM of lncRNAs in 32 (86.49%) species was less than five. However, the mean FPKM values were >12.65, 9.62, and 8.04 in the fern *S. moellendorffii*, the eudicot *M. esculenta*, and the monocot *O. punctate*, respectively (Fig. 1a, b, Table S1). Furthermore, the average median FPKM value of lncRNAs in *S. moellendorffii* was >1.69, which was far greater than that in other species, indicating that lncRNAs might have specific expression patterns and functional mechanisms in *S. moellendorffii*.

**Fig. 2:**
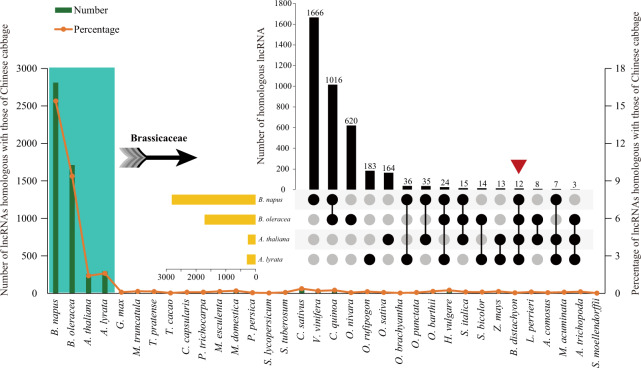
Similarity alignment of lncRNAs in Chinese cabbage and those in 36 other species. The bar chart in green indicates the number of lncRNAs in each examined species homologous to those in Chinese cabbage. The broken line in orange indicates the percentage of lncRNAs homologous to those in Chinese cabbage for each examined species. The Venn diagram shows the common and specific lncRNAs in four Brassicaceae species homologous to those in Chinese cabbage

In addition, we conducted a similarity alignment and conservation analysis of the lncRNAs of all examined species to better understand the function and evolution of plant lncRNAs. Taking our main studied species as an example, among the 18,253 lncRNAs in Chinese cabbage, only 3955 (21.67%) were homologous with those of the examined species. Among all 36 species, relatively high sequence similarity was detected within four Brassicaceae species, and 2,811 (15.40%), 1,712 (9.38%), 292 (1.60%), and 257 (1.41%) lncRNAs in Chinese cabbage had homologs in *B. napus*, *B. oleracea*, *A. lyrata*, and *A. thaliana*, respectively (Fig. 2, Tables S2–3). Furthermore, 12 lncRNAs had homologs among these four species, indicating that they might be conserved in Brassicaceae species (Fig. 2). In other non-Brassicaceae species, the maximum homology percentage was only 0.38%; in addition, no homologous lncRNAs were detected in the moss *P. patens*, but 1 was detected in the green alga *C. reinhardtii* (Fig. 2, Table S2). Taken together, these results indicated that lncRNAs in Chinese cabbage are poorly conserved with those in other species, which is consistent with previous reports for NHCC and *Fragaria vesca*^38,47^.

### The microRNA precursors identified from lncRNAs in 37 species were unique to specific species

**Fig. 3:**
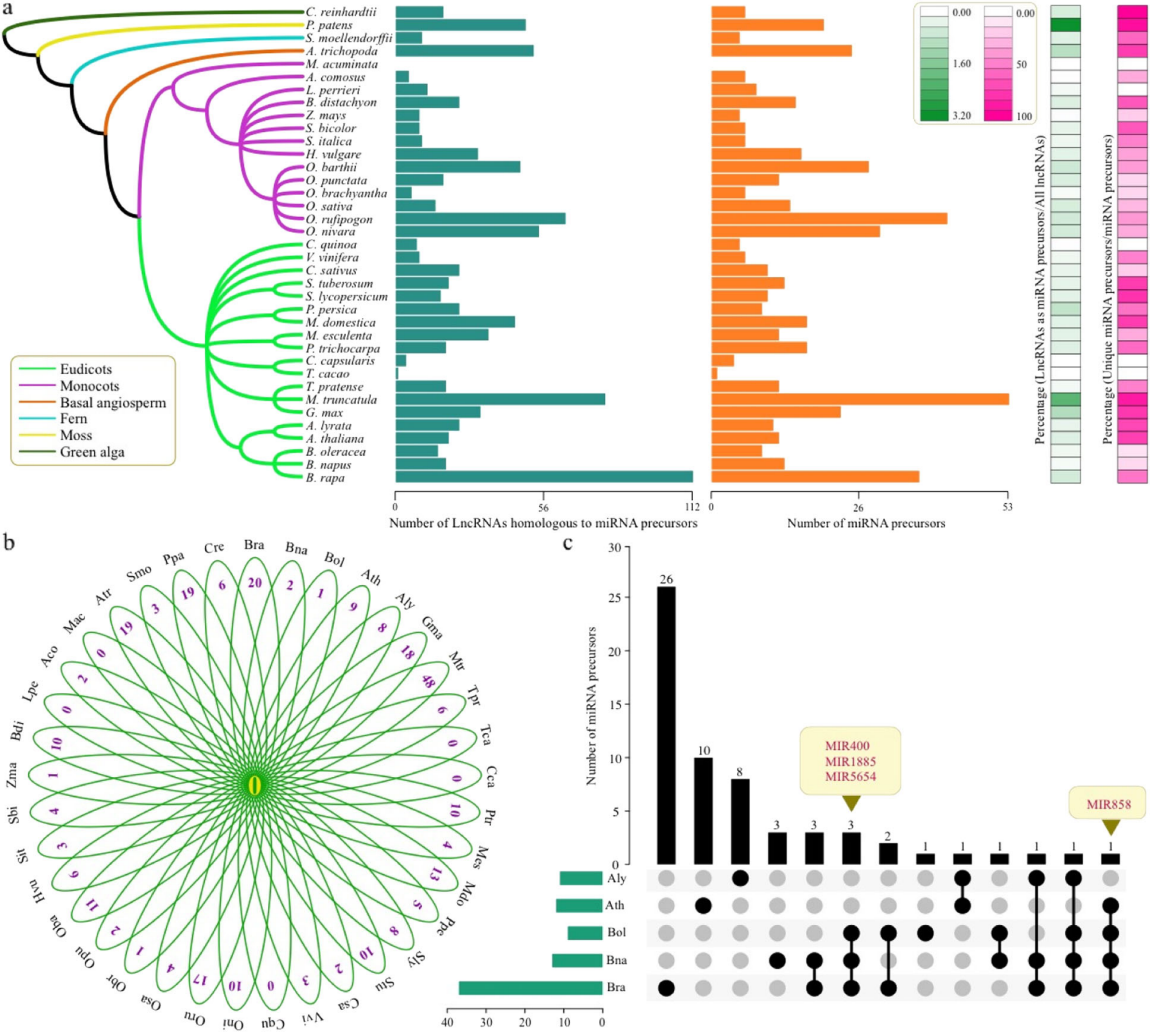
Comparative analysis of lncRNAs homologous to miRNA precursors in 37 species. **a** The green bar plot shows the number lncRNAs homologous to miRNA precursors in all examined species. The orange bar plot shows the number of homologous miRNA precursors. The green heatmap indicates the percentage of lncRNA numbers as miRNA precursors and all lncRNAs in each species. The red heatmap indicates the percentage of unique miRNA precursors and all miRNA precursors in each species. **b** Number of common and unique miRNA precursors in all 37 species. **c** Number of common and unique miRNA precursors in five Brassicaceae species

To further investigate the function of lncRNAs, we tried to identify lncRNAs that had potential interactions with miRNAs. using the BLASTN program, we identified 960 lncRNAs as miRNA precursors from 247,242 lncRNAs of 37 species (Tables S4-5). Interestingly, *B. rapa* had the most miRNA precursors (112), followed by *M. truncatula* (79) and *O. rufipogon* (64) (Fig. 3a, Table S4). However, only one miRNA precursor from lncRNAs was detected in *Theobroma* *cacao*, and no miRNAs were identified in *M. acuminata*. The average percentage of lncRNAs as miRNA precursors was 0.50%, and the range varied from 0.00% (*M. acuminata*) to 3.27% (*P. patens*) among the 37 species (Fig. 3a, Table S4).

Furthermore, we searched for common and unique miRNA precursors in the 37 species. The results showed that no common miRNA precursors were found among any of the 37 species (Fig. 3b). Among all 524 miRNA precursors, 285 (54.38%) were unique to one of the examined species (Table S4). The great number of unique miRNA precursors were detected in *M. truncatula* (48), followed by *B. rapa* (20), *Amborella* *trichopoda* (19), and *P. patens* (19) (Fig. 3b). However, no unique miRNA precursors were detected in five species: *Corchorus capsularis*, *Chenopodium* *quinoa*, *Leersia* *perrieri*, *M. acuminate,* and *T. cacao*. The percentages of unique miRNA precursors were obviously different among the 37 species, ranging from 0.00% to 100.00% (Fig. 3a, Table S4). This phenomenon suggested that lncRNAs might play a role in regulating miRNA specificity in some species.

In addition, we analyzed the common and unique miRNA precursors in five Brassicaceae species. Similarly, no common miRNA precursors were detected among the five species (Fig. 3c). However, three miRNA precursors (MIR400, MIR1885, and MIR5654) were found in three *Brassica* species: *B. rapa*, *B. oleracea*, and *B. napus* (Fig. 3c). We also found one miRNA precursor, MIR858, which was present among these three *Brassica* species and *A. thaliana*. The greatest number of unique miRNA precursors (26) were found in *B. rapa*, followed by *A. thaliana* (10), *A. lyrata* (8), *B. napus* (3), and B. *oleracea* (1).

### Identification and characterization of lncRNAs in Chinese cabbage under heat stress

**Fig. 4:**
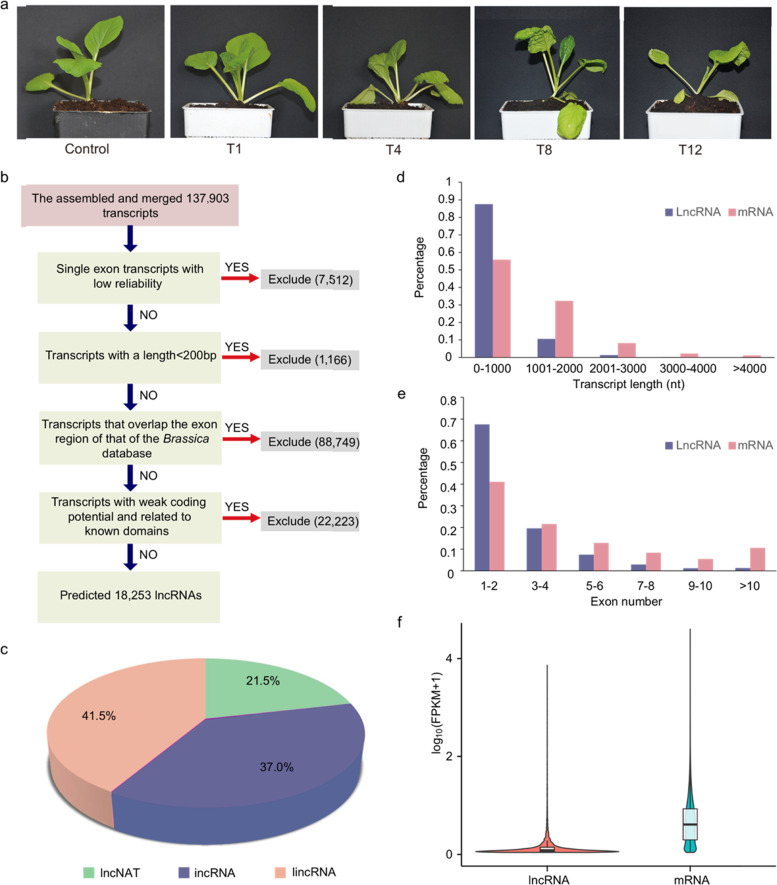
Prediction and characterization of potential lncRNAs in Chinese cabbage. **a** Phenotypes of Chinese cabbage before and after heat treatment for different times. The control is a representative plant before heating, and T1, T4, T8, and T12 denote plants that were subjected to 38 °C for 1h, 4h, 8h, and 12h, respectively. **b** The bioinformatics pipeline for the systematic identification of lncRNAs in Chinese cabbage. **c** Classification of the identified lncRNAs based on their genomic positions with protein-coding genes. **d**–**f** The distribution of transcript length, exon number, and FPKM value of both lncRNAs and mRNAs in Chinese cabbage

To identify and explore the heat-responsive lncRNAs and regulatory mechanisms of Chinese cabbage, we conducted lncRNA sequencing in this study. In the control samples, the leaves were fully turgid, while they showed different degrees of wilting at different heating times, and the effect was prominent at 4 h, 8 h, and 12 h. In addition, new leaves began to roll, and old leaves began to fall off with increased duration of stress (Fig. 4a). High-throughput sequencing was performed on samples harvested from fully expanded upper leaves under different treatment conditions to screen for potential lncRNAs that respond to heat stress in Chinese cabbage. Each sample involved three biological replications. After filtering and screening, 79.1–106.2 million clean reads were generated for each replicate, of which >66.99% of reads could be mapped to unique positions along the Chinese cabbage reference genome (Table 1). A total of 197.41 Gb were obtained, with a range from 36.34 Gb (T1) to 43.53 Gb (T8), among the different treatments. The Q30 base percentage of all the replicates was more than 93.75%, indicating the credibility of the RNA-seq data (Table 1).

**Table 1:** Statistical analysis of lncRNA sequencing data from five samples with three biological replicates in Chinese cabbage

Sample name	Replicate name	Clean reads	Clean bases (Gb)	Total clean bases (Gb)	Q30 (%)	Unique mapped reads
Control	Control-1	88,164,550	13.22	38.67	94.41	62,694,500 (71.11%)
	Control-2	86,521,924	12.98		94.52	61,446,125 (71.02%)
	Control-3	83,148,774	12.47		93.75	58,180,935 (69.97%)
T1	T1-1	79,309,130	11.90	36.34	94.02	56,587,130 (71.35%)
	T1-2	83,815,786	12.57		93.84	59,614,126 (71.13%)
	T1-3	79,157,270	11.87		94.39	57,026,412 (72.04%)
T4	T4-1	85,038,374	12.76	40.84	94.92	59,222,002 (69.64%)
	T4-2	106,167,856	15.93		94.94	74,975,222 (70.62%)
	T4-3	81,013,688	12.15		94.60	57,424,480 (70.88%)
T8	T8-1	91,647,384	13.75	43.53	95.01	63,164,281 (68.92%)
	T8-2	100,562,348	15.08		94.96	70,323,780 (69.93%)
	T8-3	98,028,886	14.70		94.87	67,817,950 (69.18%)
T12	T12-1	81,109,770	12.17	38.03	94.20	55,789,226 (68.78%)
	T12-2	84,645,330	12.70		94.35	57,825,950 (68.32%)
	T12-3	87,720,638	13.16		95.34	58,759,700 (66.99%)

In total, 137,903 transcripts were ultimately obtained from fifteen libraries, among which 18,253 transcripts were identified as lncRNAs after a series of strict screening pipelines (Fig. 4b, Table S6). The lncRNAs were distributed across 10 chromosomes in Chinese cabbage, and the largest number of lncRNAs were on chromosome A09 (Fig. S2, Table S6). The lncRNAs were subdivided into different categories according to their locations, and the majority of lncRNAs were lincRNAs, accounting for ~41.5%, followed by incRNAs (37.0%) and lncNATs (21.5%) (Fig. 4c, Table S6).

To further characterize the features of the identified lncRNAs in Chinese cabbage, the length and exon number of 18,253 lncRNAs were analyzed and compared with those of mRNAs. It was found that lncRNAs with a length of >1000 nt accounted for only 12.4%. In comparison, 44.1% of the mRNAs were >1000 nt in length (Fig. 4d, Table S6). In addition, these lncRNAs and mRNAs had a different number of exons; ~67.6% of lncRNAs contained one or two exons, whereas it was ~55.9% for the mRNAs (Fig. 4e, Table S6). The expression levels of lncRNAs and mRNAs were compared according to their FPKM values at different time points. Regardless of whether the samples were heat treated, the expression of lncRNAs was notably lower than that of mRNAs (Figs. 4f and S3). Despite the low expression levels, many lncRNAs exhibited distinct expression patterns. The number of lncRNAs detected at different treatment times ranged from 13,254 in the control to 15,174 at 12 h after heat treatment (Table S7). Interestingly, we found that with increasing heat treatment time, the number of detected lncRNAs and mRNAs also gradually increased. The correlations among the three biological replicates of each sample were examined by computing Pearson correlation coefficients (PCCs). The results showed strong correlations, with PCC values ranging from 0.8 to 1 for different replicates, indicating a high degree of repeatability (Fig. S4).

### LncRNAs and mRNAs in Chinese cabbage were selectively expressed at five stages under heat stress

Among the 18,253 lncRNAs, 9756 were expressed at all five stages (Fig. S5a). Most lncRNAs were selectively expressed at certain stages, and a considerable number of them were expressed specifically at one stage. The most stage-specific expressed lncRNAs (422) were detected at 12 h after heat treatment, whereas the fewest (265) specifically expressed lncRNAs were detected in the control (Fig. S5a). In addition, by surveying the sum of DE lncRNAs in response to heat stress at different times, a total of 1229 lncRNAs were identified as being DE in comparison with the lncRNAs in the control sample, and 88 DE lncRNAs were shared among all comparison groups (T1, T4, T8, and T12 vs the control, respectively) (Fig. S6a, Tables S8-9). The number of DE lncRNAs increased gradually with increased heating time, and upregulated lncRNAs constituted a larger proportion than the downregulated lncRNAs did in each comparison group (Fig. S6b). For mRNAs, a total of 6836 genes showed significantly differential expression compared with those in the control, of which 1086 were commonly expressed based on Venn diagrams (Figs. S5b and S6c, Tables S10-11). In addition, more downregulated genes were detected in T8 and T12 than in T1 and T4 compared with the control (Fig. S6d).

To investigate the temporal patterns of these DE lncRNAs and DE mRNAs under different stress stages, cluster analysis was employed using the STEM program. The DE lncRNAs were classified into 38 distinct profiles, each representing a group of genes presenting the same expression pattern. Among them, seven profiles were significantly enriched (*P* < 0.05) and were further divided into four clusters with different expression trends according to the background color (Fig. S6e). The DE mRNAs for each treatment compared to the control also clustered into distinct profiles based on their expression patterns, and a total of 11 expression profiles were significantly enriched, which was more than that of DE lncRNAs (Fig. S6f).

### Interaction network construction of *cis*- or *trans*-regulated protein-coding genes of DE lncRNAs

**Fig. 5:**
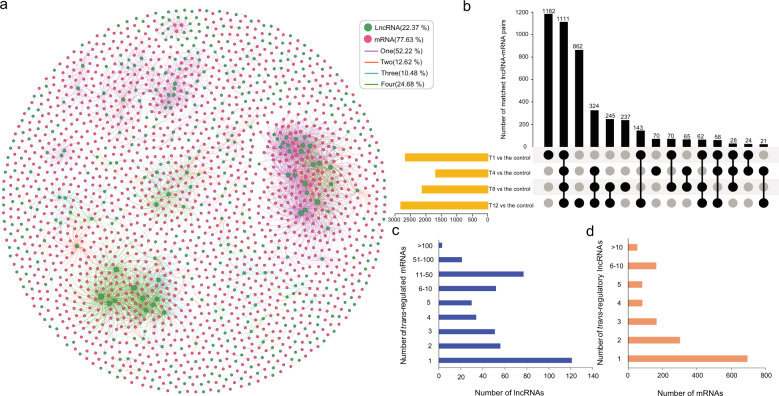
Interactions between DE lncRNAs and their trans-regulated DE mRNAs in Chinese cabbage. **a** A combined DE lncRNA-mRNA interaction network of the four comparisons (T1, T4, T8, and T12 vs the control, respectively) constructed by Gephi software. The nodes represent lncRNAs (green) and mRNAs (red), and the edges represent the interactions between lncRNAs and mRNAs. The node size corresponds to the number of interacting mRNAs or lncRNAs, and the edge color denotes the number of comparisons where different lncRNA-mRNA connections are present. **b** Venn diagram showing the number of common and specific matched lncRNA-mRNA pairs in the different comparisons. The black and gray dots represent the lncRNA-mRNA pairs present and absent in the comparisons, respectively. **c** Number of DE mRNAs regulated by DE lncRNAs. **d** Number of DE lncRNAs that have potential *trans*-regulatory effects on DE mRNAs

LncRNAs have been found to act through *cis*- and *trans*-acting modes to regulate the expression of proximal and distal protein-coding genes^27,28^. In our study, the *trans*-regulated genes were predicted by coexpression analysis based on the expression level of DE lncRNAs and DE mRNAs among samples. The regulatory network was constructed by Gephi software (Fig. 5a). Among 1229 DE lncRNAs, 445 were predicted to have potential *trans*-acting effects on 1544 DE mRNAs in 4502 matched pairs. Among them, 1111 *trans*-regulatory matches (24.68%) were shared among all comparisons, and 2351 (52.22%) were exclusively expressed in only one comparison group (Fig. 5a, b, Table S12). The T12 vs control group had the most matched pairs, whereas the most abundant stage-specific lncRNA-mRNA interactions were detected in the T1 vs control group (Fig. 5b). The lncRNAs were regulated via 1–115 mRNAs in this mode, of which >27% of lncRNAs were coexpressed together with only 1 mRNA, and three lncRNAs (*LNC_011542*, *LNC_007838*, and *LNC_016696*) had >100 *trans*-regulated targets (Fig. 5c, Tables S13–1). More than 44% of mRNAs corresponded to only one lncRNA, and 53 mRNAs (3.4%) were potentially regulated by >10 lncRNAs (Fig. 5d, Tables S13–2).

**Table 2:** Key lncRNAs identified in this study that are related to heat response in Chinese cabbage

lncRNA_ID	Potential target genes	Number of potential target genes	Activity mode^a^
*LNC_007838*	*HSP*s and *HSP-related* genes	21	*cis* or *trans*
*LNC_016696*	*HSP*s and *HSP-related* genes	21	*cis* or *trans*
*LNC_010992*	*HSP*s and *HSP-related* genes	17	*trans*
*LNC_003028*	*HSP*s and *HSP-related* genes	16	*trans*
*LNC_005395*	*HSP*s and *HSP-related* genes	13	*trans*
*LNC_009853*	*HSP*s and *HSP-related* genes	13	*trans*
*LNC_003977*	*HSP*s and *HSP-related* genes	11	*trans*
*LNC_005895*	*HSP*s and *HSP-related* genes	11	*cis* or *trans*
*LNC_006136*	*HSP*s and *HSP-related* genes	11	*trans*
*LNC_007840*	*HSP*s and *HSP-related* genes	7	*cis* or *trans*
*LNC_015255*	*HSPs*	7	*cis* or *trans*
*LNC_004890*	*PYR*/*PYL*s	6	*trans*
*LNC_005790*	*PYR*/*PYL*s	6	*trans*
*LNC_005338*	*PYR*/*PYL*s	5	*trans*
*LNC_005590*	*PYR*/*PYL*s	5	*trans*
*LNC_017421*	*PYR*/*PYL*s	5	*trans*
*LNC_001877*	*PYR*/*PYL*s	4	*trans*
*LNC_006162*	*PYR*/*PYL*s	4	*trans*
*LNC_006421*	*PYR*/*PYL*s	4	*trans*
*LNC_011542*	*PYR*/*PYL*s	4	*trans*
*LNC_010992*	*clpB1*	1	*cis*
*LNC_013535*	*DHR18*	1	*cis*
*LNC_000609*	*slu7*	1	*cis*
*LNC_002021*	*MLP*-like genes	1	*cis*
*LNC_000283*	*MLP*-like genes	1	*cis*
*LNC_014195*	*MLP*-like genes	1	*cis*

^a^For *trans*-acting mode, lncRNAs that might regulate more than ten *HSP* genes and more than three *PYR*/*PYL* genes were selected.

LncRNAs are known to preferentially regulate genes located in close proximity to their transcription sites. Therefore, the proximal protein-coding genes located within a genomic window of 100 kb of lncRNAs were screened as their target genes for *cis* activity. Among the 1,229 DE lncRNAs, 1,027 were computationally predicted to have potential *cis*-acting effects on 2,651 DE mRNAs in 4,090 matched pairs, while only 108 matched pairs (2.64%) were shared among all comparisons (Fig. S7, Table S14). In the T12 vs control group, the number of total matched lncRNA-mRNA connections and stage-specific lncRNA-mRNA interactions were the highest (Fig. S7b). Among all the matches, more than 75% of lncRNAs could regulate 1–5 target genes, and 11 lncRNAs (1.07%) could target up to 10 mRNAs (Fig. S7c, Tables S15–1). More than 67% of the mRNAs corresponded to only one lncRNA, and the expression of only 5 genes (0.19%) was *cis*-regulated by more than 10 lncRNAs (Fig. S7d, Tables S15–2).

Furthermore, we combined lncRNA-mRNA interactions with respect to both *cis*- and *trans*-regulatory modes. In total, 86 interactions involving 81 DE lncRNAs and 86 DE mRNAs were eventually found to be both coexpressed and were less than 100 kb apart (Table S16). Interestingly, 71 connections were sense-antisense (SA) pairings of transcripts, and the PCC value of each SA pair was greater than 0.95, with a *p* value <0.01, indicating that these lncRNAs were positively correlated with their cognate sense genes (Table S16).

### ceRNA network analysis revealed the critical miRNA response to heat stress

**Fig. 6:**
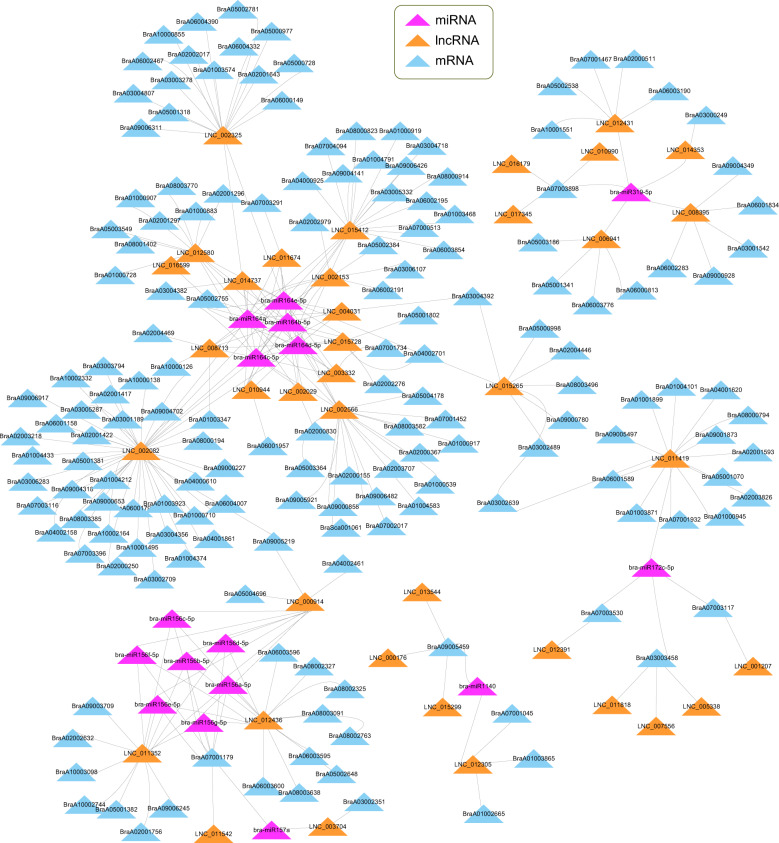
Heat response ceRNA network of Chinese cabbage. Heat response ceRNA network of miRNAs (pink), lncRNAs (orange), and target mRNAs (blue) in Chinese cabbage

One of the most important functions of lncRNAs is to act as ceRNAs, which can act as decoys for miRNAs to competitively inhibit their interaction with target mRNAs. Therefore, a ceRNA network was constructed to predict the interaction relationships among lncRNAs, miRNAs and mRNAs in the heat stress response of Chinese cabbage (Fig. 6).

Here, the DE lncRNAs and mRNAs of the coexpression network were used as prediction libraries of ceRNA and target mRNA targets of miRNAs, respectively. A total of 157 mature miRNAs obtained from the miRbase database were used as bait to predict ceRNAs and target mRNAs. First, RNAhybrid software predicted that 75 miRNAs could be decoyed by 104 lncRNAs, forming 218 lncRNA-miRNA pairs (Table S17). Thirty-one miRNAs were then predicted to target 23 mRNAs, forming 37 miRNA-mRNA pairs (Table S18). Finally, the above two relation data sets were cross-referenced, and the lncRNA and mRNA pairs sharing the same miRNA were selected to construct the ceRNA network (Fig. 6, Table S19).

The ceRNA network comprised 38 lncRNAs, 16 miRNAs, and 167 mRNAs (Fig. 6, Table S19).

Interestingly, we found that seven miRNAs were members of the bra-miR156 type, and five miRNAs were members of the bra-miR164 type. These two types of miRNAs accounted for 75% of the total miRNAs of the ceRNA network, indicating that they might play important roles in regulating heat tolerance by interacting with lncRNAs and mRNAs in Chinese cabbage. This conclusion was consistent with those of previous reports on the function of these two miRNAs^48–50^. Therefore, lncRNAs interacting with these miRNAs might also play an important regulatory role in the heat tolerance of Chinese cabbage.

### Functional enrichment analysis of genes regulated by DE lncRNAs via *cis-* and *trans*-regulatory activity

**Fig. 7:**
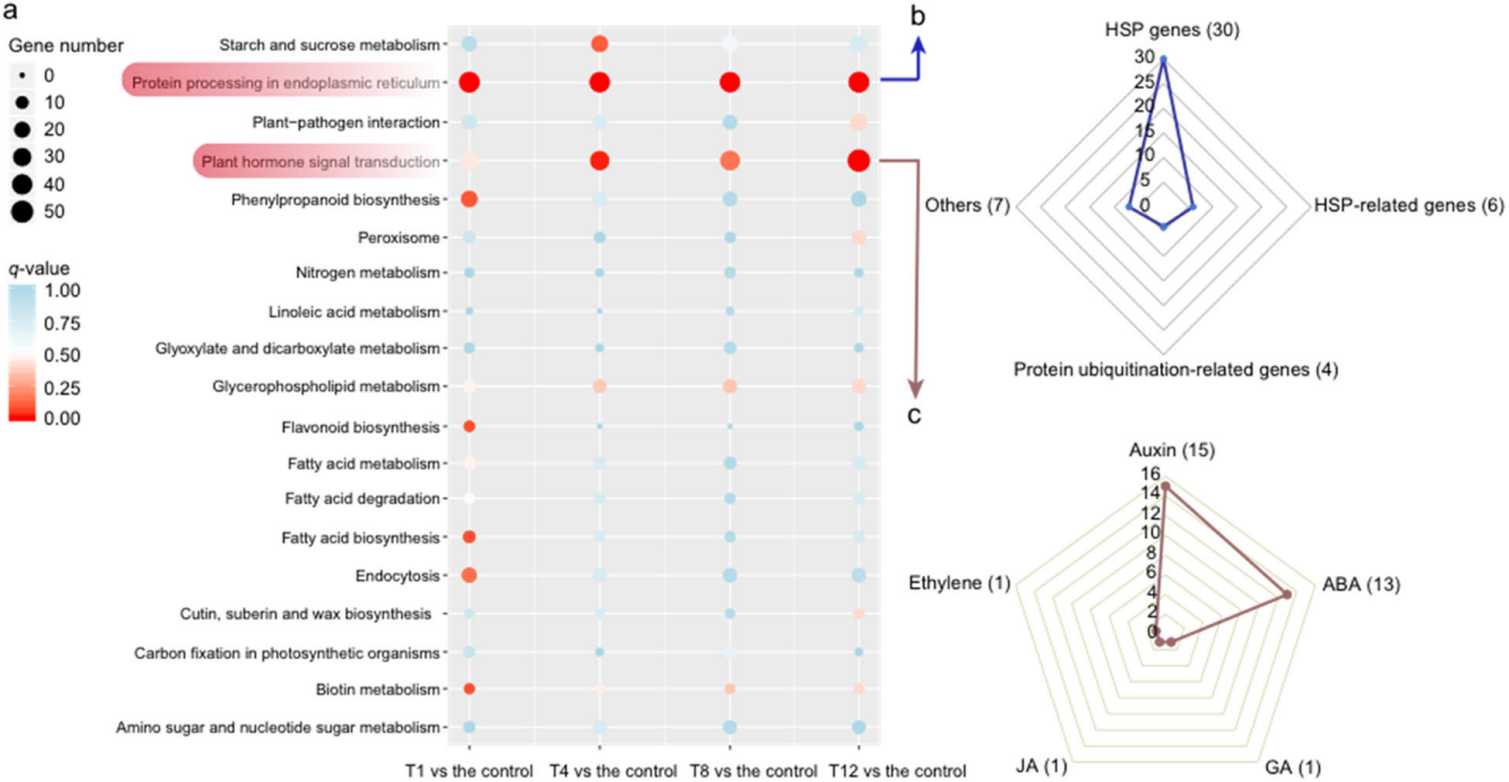
KEGG enrichment analysis of DE lncRNA-trans-regulated genes in different comparison groups. **a** Combination of the top 10 enriched pathways of each comparison. The size of each dot indicates the number of enriched genes in this pathway, and the color of each dot corresponds to different *q* value ranges. **b**–**c** Number of genes in different functional groups in the “protein processing in endoplasmic reticulum” and “plant hormone signal transduction” pathways. ABA, GA, and JA represent abscisic acid, gibberellin, and jasmonic acid, respectively

To further elucidate the roles of lncRNAs in response to heat stress at different stages of the treatment, the potential *cis*- and *trans*-regulated genes of all DE lncRNAs in the T1, T4, T8, and T12 vs control groups were subjected to GO and KEGG enrichment analysis. However, neither GO terms nor KEGG pathways were found to be significantly enriched in any comparison group for *cis*-regulation. Regarding *trans-*regulation, the potential targets were significantly enriched in seven biological process categories and one molecular function category. Those in the T1, T4, and T8 vs control groups were all highly enriched for the “cell morphogenesis” and “cellular component morphogenesis” subcategories (Table S20). In addition, the *trans*-regulated genes were assigned to 1–2 highly enriched pathways in each comparison (Table S21). The common enriched category in the four comparisons was “protein processing in endoplasmic reticulum” (Fig. 7a, Table S21). “Plant hormone signal transduction” was another significantly enriched pathway in the T4 vs control and T12 vs control groups. Furthermore, to understand the role of DE lncRNAs in response to heat, we divided all DE genes involved in these two KEGG pathways into different groups according to their functional annotations (Fig. 7b, c).

### Protein processing in the endoplasmic reticulum pathway

**Fig. 8:**
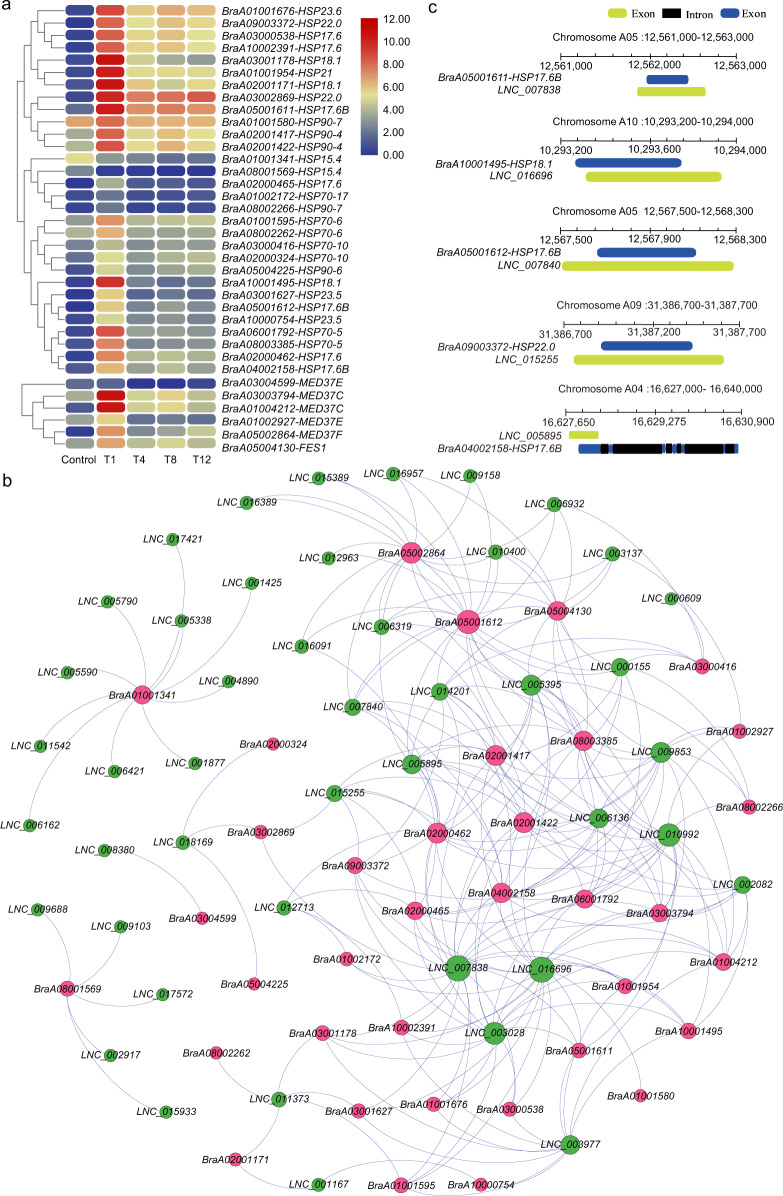
Analysis of lncRNAs and their target HSPs or HSP-related genes enriched in the “protein processing in endoplasmic reticulum” pathway. **a** Heatmap showing the expression pattern of *HSP*s and HSP-related genes. **b** Interaction network of lncRNAs together with *HSP*s and HSP-related genes. The nodes represent lncRNAs (green) and mRNAs (red), and the node size corresponds to the number of interacting mRNAs or lncRNAs. **c** Genomic locations of five *HSP* genes and their complementary lncRNAs

The “protein processing in endoplasmic reticulum” pathway involved 47 DE genes in the four comparison groups, of which 30 were annotated as encoding heat-shock proteins (HSPs) (Fig. 7b, Table S22). HSPs are known to play master roles in heat stress and act as molecular chaperones by reestablishing functional protein conformations^51–53^. Further investigation of the expression of *HSP*s showed that nearly all *HSP*s displayed extremely low expression levels in the control but that their expression dramatically increased at 1 h after heat treatment (Fig. 8a). The results demonstrated that a high proportion of *HSP*s regulated by DE lncRNAs exhibited a common expression pattern, and their predominant response to heat stress occurred at an early stage. Among HSP-related genes, five were annotated as mediators of RNA polymerase II transcription subunit 37 (MED37) (Table S22). Correspondingly, the expression patterns of four *MED37* genes were similar to those of most *HSP*s (Fig. 8a).

Expression of the *HSP*s and *HSP*-related genes was *trans*-regulated by 45 lncRNAs, forming 235 connections in the network (Figs. 8b and S8). The network indicated that the expression of seven genes was *trans*-regulated by >10 lncRNAs. Specifically, the expression of *BraA05001612* was simultaneously regulated by 18 lncRNAs (Figs. 8b and S9a, Tables S23–1). In addition, 9 lncRNAs could *trans*-regulate the expression of more than 10 *HSP*s and *HSP*-related genes, of which *LNC_007838* and *LNC_016696* could interact with up to 21 targets, implying key regulatory roles of these HSPs in the heat response (Figs. 8b and S9b, Tables S23–2). Furthermore, chromosome location analysis showed that five of the abovementioned lncRNAs, *LNC_007838*, *LNC_016696*, *LNC_007840*, *LNC_015255*, and *LNC_005895*, were in the antisense orientation and overlapped with their corresponding *HSP* genes (Fig. 8c, Table S16). This phenomenon indicated that these five lncRNAs might regulate the expression of their cognate sense *HSP*s by a *cis*-acting mode.

Moreover, four genes enriched in this pathway were identified as being associated with protein ubiquitination, and the expression of *BraA01004433* and *BraA06003499* peaked 1 h after heat treatment (Fig. S10a, Table S22). The interaction network showed that the expression of these four genes is regulated by 22 lncRNAs. Among the expression of the four genes, that of *BraA01004433* was regulated by 13 lncRNAs (Fig. S10b). It is known that polyubiquitination often leads to the degradation of target proteins by the 26 S proteosome^54,55^. Therefore, we speculated that lncRNAs might regulate genes involved in the ubiquitin system, especially by regulating the expression of *BraA01004433*, to more effectively remove the deleterious and denatured proteins caused by high temperature.

### Plant hormone signal transduction pathway

**Fig. 9:**
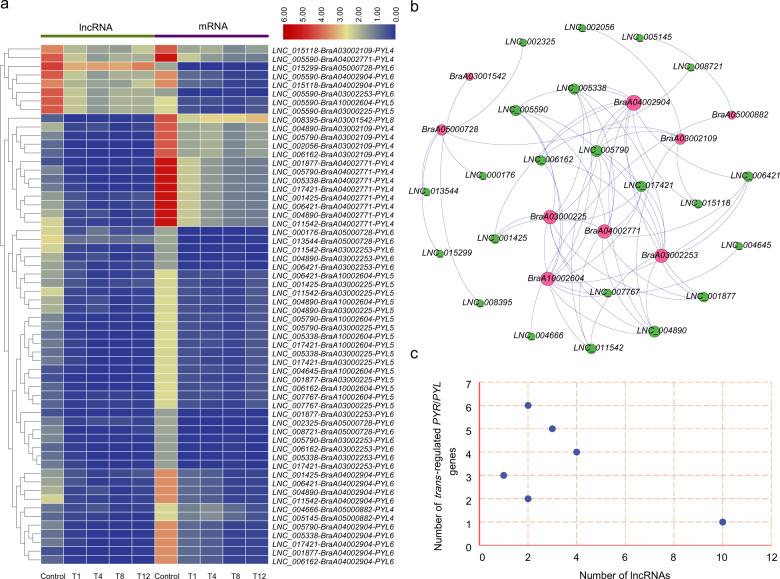
Expression patterns and interaction network of lncRNAs and their trans-regulated PYR/PYL family genes enriched in the ‘plant hormone signal transduction’ pathway. **a** Heatmaps showing the expression patterns of lncRNAs and their *trans*-regulated PYR/PYL family genes. **b** Interaction network between lncRNAs and *PYR/PYL* family genes. The nodes represent lncRNAs (green) and mRNAs (red), and the node size corresponds to the number of interacting mRNAs or lncRNAs. **c** Number of *PYR/PYL* family genes *trans*-regulated by each lncRNA

Plant hormones are reported to play important roles in regulating responses to heat stress^44,56–58^. In our study, a total of 31 DE genes was enriched in the “plant hormone signal transduction” pathway. Superficially, 15 DE genes were related to auxin, followed by abscisic acid (ABA, 13), gibberellin (GA, 1), ethylene (1), and jasmonic acid (JA, 1) (Fig. 7c, Table S24). Through comprehensive assessment of the expression of these genes, it was remarkable that 9 genes identified as members of the ABA receptor pyrabactin resistance (*PYR*)/*PYR*-like (*PYL*) family exhibited a common expression trend. After 1 h of heat treatment, the expression of *PYR*/*PYL*s decreased dramatically, while the change in later stages of treatment was not significant (Figs. 9a and S11a, Table S24).

ABA is a major hormone that plays a key role in the resistance to multiple abiotic stresses^56,59^. Coexpression analysis demonstrated that 22 lncRNAs were highly correlated with these *PYR*/*PYL* genes and formed 60 interaction pairs (Fig. 9a). Five *PYR/PYL* genes were found to be targeted by 9–10 lncRNAs in the network (Figs. 9b and S11b, Tables S25–1). In addition, nine lncRNAs could *trans*-regulate the expression of 4–6 *PYR*/*PYL*s. Among them, *LNC_004890* and *LNC_005790* had the most target genes and could regulate 6 *PYR*/*PYL*s (Fig. 9b, c, Tables S25–2). These results suggested that the interaction network provided effective evidence for estimating gene function and that lncRNAs might be involved in triggering ABA-mediated plant heat responses through *trans-*regulation of the expression of ABA receptor genes. In addition, lncRNAs interacting with a large number of *PYR*/*PYL*s could be used as candidates for further functional analysis.

### Identification of eight genes expressed in response to stimuli via combined analysis of *cis-* and *trans-*regulatory lncRNA-mRNAs

**Fig. 10:**
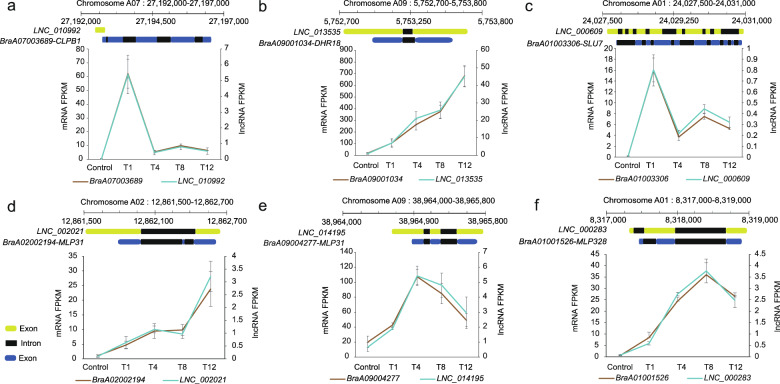
Expression coordination of lncRNAs and their *cis*-regulated neighboring genes involved with the “response to stimulus” GO term. Expression patterns and genomic locations of lncRNAs and their corresponding target genes, which were orthologous to *ClpB1* **a**, *Dehydrin Rab 18* (*DHR18*) **b**, *SLU17* **c,** and *MLP-like* genes **d**–**f** in Arabidopsis or rice. Each value is the mean ± SE of three replications (*n* = 3)

To narrow the range of critical lncRNA-mRNA pairs in the sensing of and response to heat stress in Chinese cabbage, a total of 81 DE lncRNAs and 86 DE mRNAs were identified in 86 lncRNA-mRNA pairs via combined *cis-* and *trans-*regulatory analysis (Table S16). Moreover, the target genes involved in “response to stimulus” were screened for further study according to their GO annotations. The results indicated that eight genes were involved with this term, and six of them had functional annotations (Table S26). Expression analysis showed that all six genes exhibited an upward expression trend in the early stage (T1) of heat treatment (Fig. 10).

*BraA07003689* was annotated as encoding a caseinolytic peptidase B (ClpB) protein, which, with the assistance of Dnak (HSP70), helps to remodel the structure of stress-damaged proteins from an aggregate state^60,61^. The expression trend of *BraA07003689* was consistent with that of the majority of *HSP* genes, which was expressed in response to heat stress rapidly, being substantially upregulation at 1 h under heat stress, followed by a dramatic decline in expression (Fig. 10a). *BraA07003689* was predicted to be *cis*-regulated by *LNC_010992*, which is located in the opposite strand and partially overlaps with the 3′ end of *BraA07003689* (Fig. 10a, Table S26).

The expression of the protein-coding gene *BraA09001034*, which is predicted to encode a dehydrin protein, was found to gradually increase under heat treatment (Fig. 10b). High temperature often causes cell water contents to decrease, leading to a reduction in cell size^62^. Dehydrins belong to group 2 LEA (late embryogenesis abundant) proteins, which are considered stress-related proteins and participate in the formation of the plant dehydration protective response^63,64^. The corresponding lncRNA of this dehydrin gene was *LNC_013535*, which is located in the antisense strand and encompasses the entire sequence of *BraA09001034* (Fig. 10b, Table S26).

Furthermore, the expression of *BraA01003306*, annotated as a pre-mRNA-splicing factor that is homologous to *SLU7*, quickly increased at 1 h after heat treatment (Fig. 9c). It has been reported that plants can actively use a pre-mRNA splicing mechanism to regulate the expression of stress–response genes and modulate intracellular regulatory networks^65,66^. Induction of the expression of the potential pre-mRNA-splicing factor gene *BraA01003306* in Chinese cabbage might affect the frequency and diversity of alternative splicing events of stress-responsive genes, therefore improving heat tolerance. *BraA01003306* was nested within and coexpressed together with *LNC_000609* (Fig. 10c, Table S26).

Moreover, three major latex protein (MLP)-like genes, *BraA02002194*, *BraA09004277,* and *BraA01001526*, exhibited different expression patterns (Fig. 10d–f). MLP-like proteins have been identified to respond to abiotic stress in a variety of plant species and might function by participating in the ABA signaling pathway^67–70^. The lncRNAs coexpressed with these genes, *LNC_002021*, *LNC_014195*, and *LNC_000283*, all encompassed their corresponding target mRNAs as antisense partners (Fig. 10d–f, Table S26).

Overall, by conducting a functional analysis of *cis*- and *trans*-regulated genes, we explored additional valuable lncRNAs in response to heat stress, and their potential functions were determined. This greatly enhanced our understanding of plant responses and adaptations to heat stress.

### Expression verification of lncRNAs and their potential target genes

**Fig. 11:**
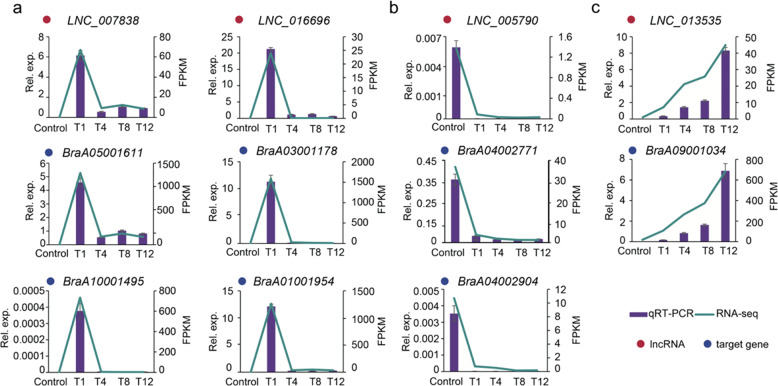
Verification of RNA-seq results via qRT-PCR. The FPKM and relative expression level of lncRNAs and their regulated mRNAs. **a** lncRNAs and target *HSP* genes. **b** lncRNAs and *PYR*/*PYL* genes. **c** lncRNAs and *Dehydrin Rab 18* (*DHR18*). For qRT-PCR, each value is the mean ± SE (*n* = 3); for RNA-seq, each value is the mean of three replications

According to our analysis, several lncRNAs were believed to play important roles in coping with heat stress in Chinese cabbage by regulating the expression of *HSP*s, HSP-related genes, and the PYR/PYL group of ABA receptor genes (Table 2). To verify the accuracy, the expression patterns of several critical lncRNAs and their potential targets were verified by quantitative real-time PCR (qRT-PCR) (Fig. 11a–c). The qRT-PCR results were consistent with those obtained from RNA-seq, suggesting that these identified lncRNAs might modulate a series of genes with different functions in response to heat stress through coexpression.

## Discussion

Sequence similarity and conservation are regarded as indicators of biological function. Our analyses showed that lncRNAs present low homology in 37 species (Fig. 2). The low sequence conservation among different species was in accordance with the findings of previous studies, which could be explained in two ways^21,22,71^. Some researchers consider that the lack of cross-species conservation of lncRNAs might indicate unimportant functions, with many lncRNAs probably being transcriptional byproducts or “transcriptional noise”^72,73^. By contrast, low conservation levels might be intrinsic to rapidly evolving lncRNAs, which form a species-specific control layer to regulate gene expression in multiple ways^74^.

Generally, lncRNAs function by regulating related genes in a variety of ways, among which they have been reported as the precursor sequences of miRNAs in several species^2,29,75,76^. In this study, we systematically compared and analyzed lncRNAs as miRNA precursors in 37 species. Our analysis showed that the number and percentage of lncRNAs as miRNA precursors among different species were very different (Fig. 3a). On the one hand, this might be owing to the incompleteness of lncRNAs in some species. On the other hand, this might also be due to the different ways in which lncRNAs act in different species.

A large number of lncRNAs have been identified to respond to different abiotic stresses in plants, but there have been no reports about them in Chinese cabbage under heat stress^39,40^. In the 21st century, extreme high-temperature events are expected to have negative impacts on crop growth and yield, severely threatening food security and sustainable agricultural development (IPCC 2014). Therefore, a deeper understanding of the complex heat response mechanism and improved heat tolerance in Chinese cabbage are of great significance. In this study, we systematically identified a total of 18,253 potential lncRNAs in Chinese cabbage by conducting next-generation sequencing, which provided an abundance of resources for future studies (Table S6). The number of identified lncRNAs was greater than that identified in nonheading Chinese cabbage^38^, which may be partially owing to the variation in plant subspecies, the influence of sequencing depth, or the experimental conditions.

The general characteristics of lncRNAs in Chinese cabbage under heat treatment are described in detail in this study. The majority of lncRNAs had a length of <1000 nt and contained only 1–2 exons (Fig. 4d, e), which was in accordance with findings of previous reports in other species^77–79^. Moreover, it was reported that lncRNAs function in a temporal-dependent manner^80,81^. In our study, a considerable number of lncRNAs were expressed exclusively at one stage. Of these lncRNAs, T12 was associated with the most stage-specific expression (422), and the lncRNAs in the control samples exhibited the least stage-specific expression (265). The differential expression of temporal-specific lncRNAs might enable them to function in a more dynamic manner.

In our study, to identify critical lncRNAs in response to heat stress and comprehensively understand the regulation of lncRNAs in plants, we carefully analyzed the coexpression relationships between lncRNAs and target genes enriched in the “protein processing in endoplasmic reticulum” pathway, which was significantly enriched in all the comparison groups (Fig. 7). By the construction and use of an interaction network, some lncRNAs were found to interact with a large number of *HSP*s or HSP-related genes involved in this pathway. Therefore, these lncRNAs might play critical roles in the heat response in Chinese cabbage (Fig. 8b, Table 2). In addition, two genes (*BraA01001341* and *BraA08001569*) encoding 15.4 kDa class V HSPs were found to negatively respond to heat stress, which was opposite the response of most *HSP*s. (Fig. 8a, Table S22). By analyzing publicly available microarray data, we found that their ortholog in Arabidopsis, *AT4G21870*, also exhibited a downward expression trend at the early stage under heat treatment. Therefore, further insight into the function of 15.4 kDa class V HSPs is necessary for a comprehensive understanding of the role of heat stress in gene expression regulation.

Phytohormones play pivotal roles in the regulation of physiological and molecular responses in plants under various abiotic stress conditions^56,82^. ABA is a major hormone involved in the plant defense response and contributes to plant thermotolerance^59,83^. Under short-term heat stress, the ABA content in secretions of bean plants was shown to increase obviously^84^. In *Brassica juncea*, soaking seeds in 0.5 μM ABA effectively alleviated the negative effects of heat stress^85^. In our work, the expression of nine *PYR/PYL* genes encoding ABA receptors decreased significantly after heat treatment, and 22 lncRNAs might *trans*-regulate their expression (Fig. 9). The downregulation of *PYR*/*PYL*s was consistent with the findings of a previous study in Arabidopsis, indicating that the upregulation of *PYR*/*PYL*s was not required for activating ABA signaling^86^. Previous studies have identified several lncRNAs whose expression is correlated with ABA. For example, drought-induced lncRNA (*DRIR*) was found to positively modulate drought and salt stress via the ABA-mediated pathway in Arabidopsis^87^. The expression of the lncRNA *BoNR8* is activated by ABA treatment in cabbage^88^. Our study identified 22 lncRNAs coexpressed together with 9 *PYR*/*PYL* genes encoding ABA receptors, and some lncRNAs, such as *LNC_004890* and *LNC_005790*, could interact with 6 *PYR*/*PYL*s. These findings improved our understanding of the ABA-mediated thermotolerance pathway, and these candidate lncRNAs could be used in future studies (Table 2).

It has been reported that lncRNAs are usually located next to the genes that they regulate^78^. For example, *COLDAIR* and *COOLAIR* are transcribed from the *FLC* locus and can repress the expression of *FLC*, therefore participating in Arabidopsis vernalization^5,89–91^. In addition, *lncRNA16397* could induce the expression of adjacent *SlGRX* genes in tomato, thereby reducing reactive oxygen damage and improving tolerance to *Phytophthora infestans*^92^. In our research, 86 DE lncRNA-mRNA pairs located within a genomic window of 100 kb were coexpressed (Table S16). Moreover, six of them were involved with the “response to stimulus” GO term (Figs. 8c, 10), indicating that these lncRNAs might play critical roles in the heat tolerance of Chinese cabbage by regulating their target genes. In addition, in the 86 matched pairs, 70 were SA pairs of transcripts. In Arabidopsis, the expression trend of the vast majority of potential regulatory lncNATs was more likely to be similar to that of their associated sense genes^93^. Moreover, ~40% of the *cis*-NATs were associated with a change in sense transcript levels during muscle development of pigs, and ~80% of them exhibited common expression patterns^94^. Likewise, each SA pair of transcripts that were coexpressed displayed a similar expression trend in our study (Table S16). Thus, lncNATs might have a stronger tendency to have a positive correlation expression pattern with their overlapping sense genes.

In summary, we conducted a comparative analysis of 247,242 lncRNAs among 37 species and identified 960 lncRNAs as miRNA precursors. Furthermore, we also performed comprehensive analyses of lncRNAs in Chinese cabbage. This study expands our knowledge of lncRNAs involved in the heat stress response by identifying DE lncRNAs and conducting *cis*- and *trans*-functional analyses in Chinese cabbage. The critical lncRNAs identified in our study provide valuable information for heat-responsive lncRNA collection in Chinese cabbage and provide a rich resource for further investigating the biological functions of lncRNAs in plants.

## Materials and methods

### Comparative analysis of lncRNAs among 37 species

The lncRNA data sets of 36 species were downloaded from the CANTATAdb 2.0 database (http://cantata.amu.edu.pl/index.html). The lncRNAs of Chinese cabbage were obtained using lncRNA sequencing in this study. Perl scripts were used to extract and statistically analyze the lncRNA number, length, expression, and exon number from the above data sets. A comparative plot of these lncRNA characteristics was constructed using the iTOL program (https://itol.embl.de/)^95^. The phylogenetic relationship of the 37 species was determined via the NCBI taxonomy website (https://www.ncbi.nlm.nih.gov/taxonomy)^96^. The similarity and conservation of the lncRNAs were determined using BLAST software (*E* value <1e-5), and the ggviolin function of ggpubr and digest libraries in the R program were used to construct violin plots (http://rpkgs.datanovia.com/ggpubr/reference/ggviolin.html). We also used BLASTN to compare all lncRNAs with the content of the miRBase database (Release 22.1), with an *E* value <1e-5, to check whether lncRNAs were miRNA precursors^97^.

### Plant materials and heat treatment

The genome sequencing material of Chiifu-401-42 Chinese cabbage was used in this study. Sterile seeds were sown in pots after germination and subsequently grown in a growth chamber under identical conditions (16 h day/8 h night photoperiod, 25 °C/18 °C day/night temperature regimen). After they had produced 4~5 leaves, seedlings with similar growth states were subjected to 38 °C for 1 h, 4 h, 8 h, or 12 h. The seedlings under different treatment times were deemed T1, T4, T8, and T12, and those not subjected to heat treatment were considered control samples (Fig. 1a). After heat stress treatment, the leaves were collected, frozen in liquid nitrogen immediately, and then stored at −80 °C until use.

### RNA isolation, library preparation, and sequencing

Total RNA was extracted from the samples using RNAiso Plus (Takara) according to the manufacturer’s protocol. After checking the RNA quantity and integrity, a total of 2 μg of RNA per sample was used for ribosomal RNA removal by an Epicentre Ribo-zero™ rRNA Removal Kit (Epicentre, USA). Sequencing libraries were generated using rRNA-depleted RNA via a NEBNext^®^ Ultra™ Directional RNA Library Prep Kit for Illumina^®^ (NEB, USA) following the manufacturer’s instructions. The quality of the libraries was then assessed on the Agilent 2100 system. Finally, the libraries were sequenced on an Illumina HiSeq 2500 platform. Library construction and lncRNA sequencing were performed by Novogene Cooperation (Beijing, China).

### Read mapping and lncRNA identification

Raw reads obtained from lncRNA sequencing were first processed to remove adaptors and low-quality reads. The remaining clean reads were subsequently aligned to the Chinese cabbage genome (http://brassicadb.org/brad/) using HISAT2 (v2.1.0)^98^. The mapped reads were then assembled by StringTie software (v2.1.1)^99^. The final transcripts were generated with the Cuffmerge tool to merge the transcripts obtained from each sample and remove the transcripts whose chain direction was uncertain^100^.

Based on the structural characteristics of lncRNAs and functional characteristics of nonencoded proteins, a series of strict screening pipelines was applied, as shown in Fig. 1b. For the exon filter, single exon transcripts with low reliability were removed. For size selection, transcripts with lengths shorter than 200 bp were excluded. Cuffcompare software was then used to screen and remove the transcripts that overlapped with the exon regions of genes of Chinese cabbage^101^. Finally, the coding-noncoding index (CNCI) and Coding Potential Calculator (CPC) programs were used to evaluate the coding potential of the transcripts, and only transcripts that passed the protein-coding-score test were used for subsequent analysis^102,103^. We also translated each transcript into all three possible frames and searched those sequences against the content of the Pfam database (https://pfam.xfam.org/), ensuring that our candidate set of lncRNAs did not contain any of the known protein family domains. The final determined lncRNAs were classified into several categories based on their genomic localization.

### Analysis of DE mRNAs and lncRNAs

The expression level of the transcripts was quantified using StringTie software after screening and identification of lncRNAs, reported as fragments per kilobase of transcript per million mapped reads (FPKM)^99^. The DE mRNAs and lncRNAs were determined by the Ballgown program^104^. In our study, the DE mRNAs and lncRNAs were defined as mRNAs or lncRNAs that were DE in at least one treatment compared with control treatment (*q* value <0.05). A heatmap and Venn diagrams of gene expression were generated by TBtools software^105^. To examine the expression patterns of DE mRNAs and lncRNAs under different treatment stages, STEM software (Carnegie Mellon University, USA) was used to cluster the DE genes and lncRNAs based on their expression patterns^106^. The maximum number of model profiles was set to 40, and the other settings used the default parameters.

### Prediction, functional enrichment, and interaction network construction

The heat-responsive lncRNAs were predicted to function by regulating the expression of the prospective target genes in a *cis*- or *trans*-acting manner. The protein-coding genes within 100 kb upstream or downstream of the lncRNAs were screened and removed as their target genes for *cis* action. The PCC was used to analyze the correlations between lncRNAs and mRNAs in samples at five different treatment stages. The lncRNA-mRNA pairs were considered to be coexpressed when the |PCC | was >0.95 and the *p* value was <0.01, and the mRNA was predicted to act in *trans* on the corresponding lncRNA genes.

To investigate the potential functions of the DE lncRNAs, their *cis*- and *trans*-regulated genes were further analyzed by GO annotations using the GOseq R package^107^. The GO terms with a *q* value <0.05 were thought to be significantly enriched. In addition, KOBAS software was used for Kyoto Encyclopedia of Genes and Genomes (KEGG) pathway enrichment analysis of the target genes^108^. To identify critical lncRNAs associated with heat tolerance, an interaction network comprising DE lncRNAs and DE mRNAs was constructed by Gephi (v0.8.2) software based on *cis*- or *trans*-regulation^109^.

### ceRNA network construction

The mature miRNA sequences of *B. rapa* were downloaded from miRBase (http://www.mirbase.org/, Release 22.1)^97^. The coexpression of DE lncRNAs and mRNAs was used to construct prediction libraries of ceRNAs and target mRNAs of miRNAs, respectively. The ceRNAs for the *B. rapa* miRNAs were predicted by the RNAhybrid program^110^. The main parameters were as follows, according to previous reports^30,31^: (i) the minimum free energy was <−25 kcal/mol, with a *p* value <0.05; (ii) bulges were permitted only at the 9th to 12th positions of the 5′ end of the miRNA sequence, and the bulge should comprise 2–4 bases; (iii) G/U pairs were allowed in the miRNA and lncRNA pairing region, and perfect pairing was required at the 2nd to 8th positions of the 5′ end of the miRNA sequence; and (iv) except for the bulge, no more than four mismatches were allowed in the lncRNA and miRNA pairing regions.

Target mRNAs of miRNAs were predicted using the psRNATarget program^111^. The following parameters were used: (i) maximum expectation < =3; (ii) maximum energy to unpair the target site ≤25; and (iii) length for complementarity scoring (HSP size) ≥20. A lncRNA-miRNA-mRNA network was subsequently constructed using Cytoscape v3.7.2 software^112^.

### Validation by real-time quantitative PCR

To validate the lncRNA sequencing results, several critical lncRNAs and their potential target genes were selected for qRT-PCR analysis. For reverse transcription PCR, first-strand cDNA was synthesized using a PrimeScript™ II 1st Strand cDNA Synthesis Kit (Takara, Dalian, China), according to the manufacturer’s instructions. The synthesized cDNA was then subjected to quantitative analysis in a CFX96™ Real-Time System (C1000™ Thermal Cycler, Bio-Rad) in conjunction with a SYBR Premix Ex Taq^TM^ II Kit (TaKaRa). Three biological replicates were included for each sample. The constitutively expressed *Actin* gene was used as the internal housekeeping gene to standardize the results. The primer pairs used are listed in Table S27.

## Supplementary information

Supplementary Figures 1–11

Supplementary tables 1-27

## Data Availability

The RNA-seq data sets of this study have been deposited in the Genome Sequence Archive in the BIG Data Center, Beijing Institute of Genomics (BIG), Chinese Academy of Sciences, under accession number CRA002707, which is publicly accessible at http://bigd.big.ac.cn/gsa. All the materials and related data in this study are available upon request.
