# Supplementary material for: Comparative analysis of long noncoding RNAs in angiosperms and characterization of long noncoding RNAs in response to heat stress in Chinese cabbage
Source: Hortic Res. 2021 Mar 1;8:48. doi: 10.1038/s41438-021-00484-4 (PMC7917108; doi:10.1038/s41438-021-00484-4)
Supplement: Supplementary file 1 — Supplementary Figures 1–11 [file 41438_2021_484_MOESM1_ESM.docx]

**Supplementary Figures 1-11**


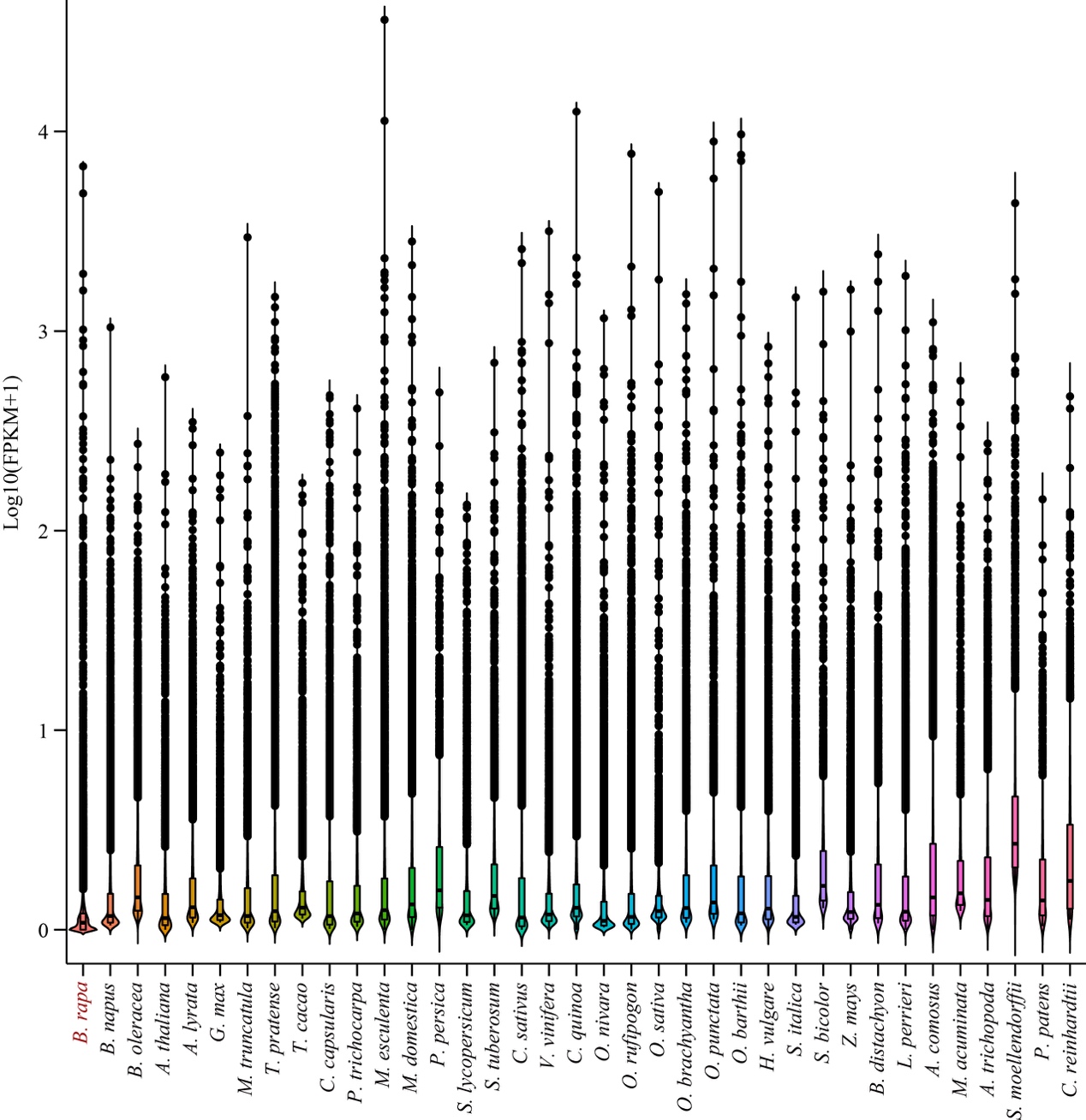


**Figure S1. The violin and boxplot plot of log_10_ (FPKM+1) for lncRNAs in 37 species.**


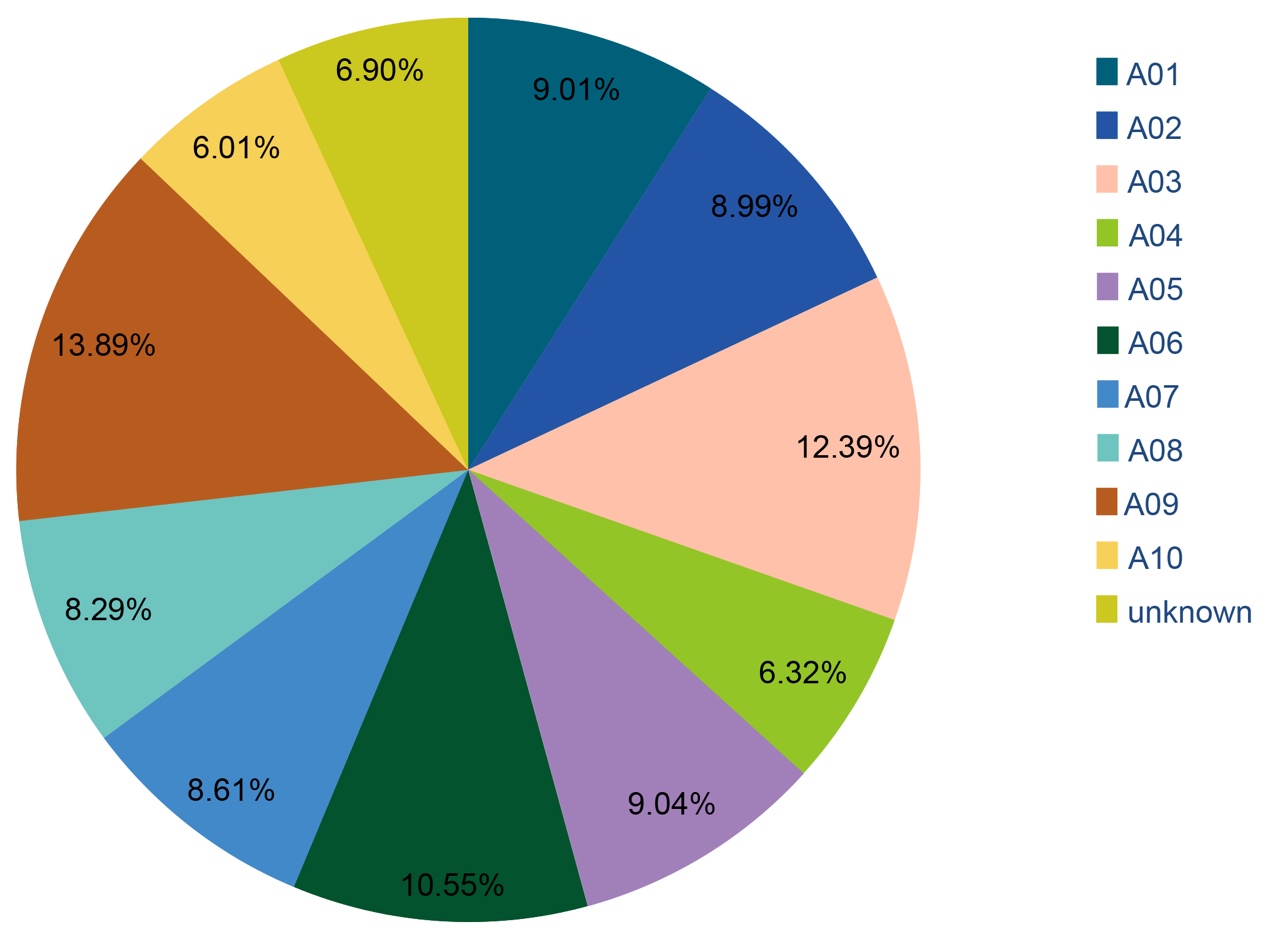


**Figure S2. The percentage of lncRNAs on each chromosome in Chinese cabbage.**


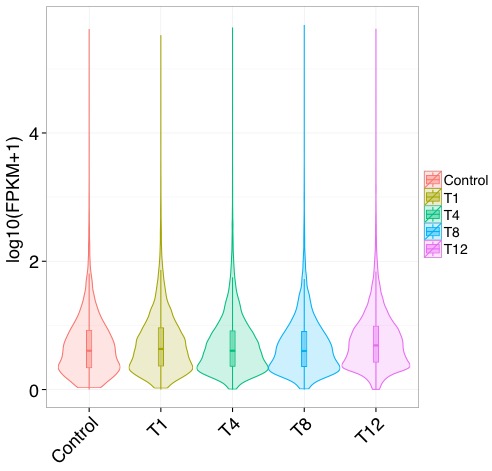


**Figure S3. The FPKM values of lncRNAs in control and each heat treatment stages under 38℃ for 1h (T1), 4h (T4), 8h (T8), and 12h (T12).**

**Figure S4. The Pearson correlation analysis among all 15 libraries. The control and each heat treatment (T1, T4, T8, and T12) with three replicates.**


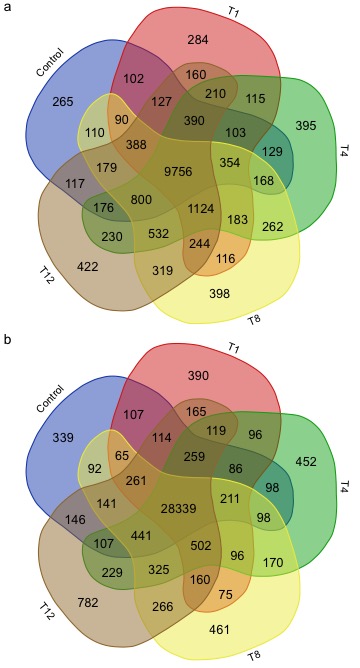


**Figure S5. Venn diagrams show the number of common and specific lncRNAs and mRNAs among different treatment stages.** (a) lncRNAs. (b) mRNAs.


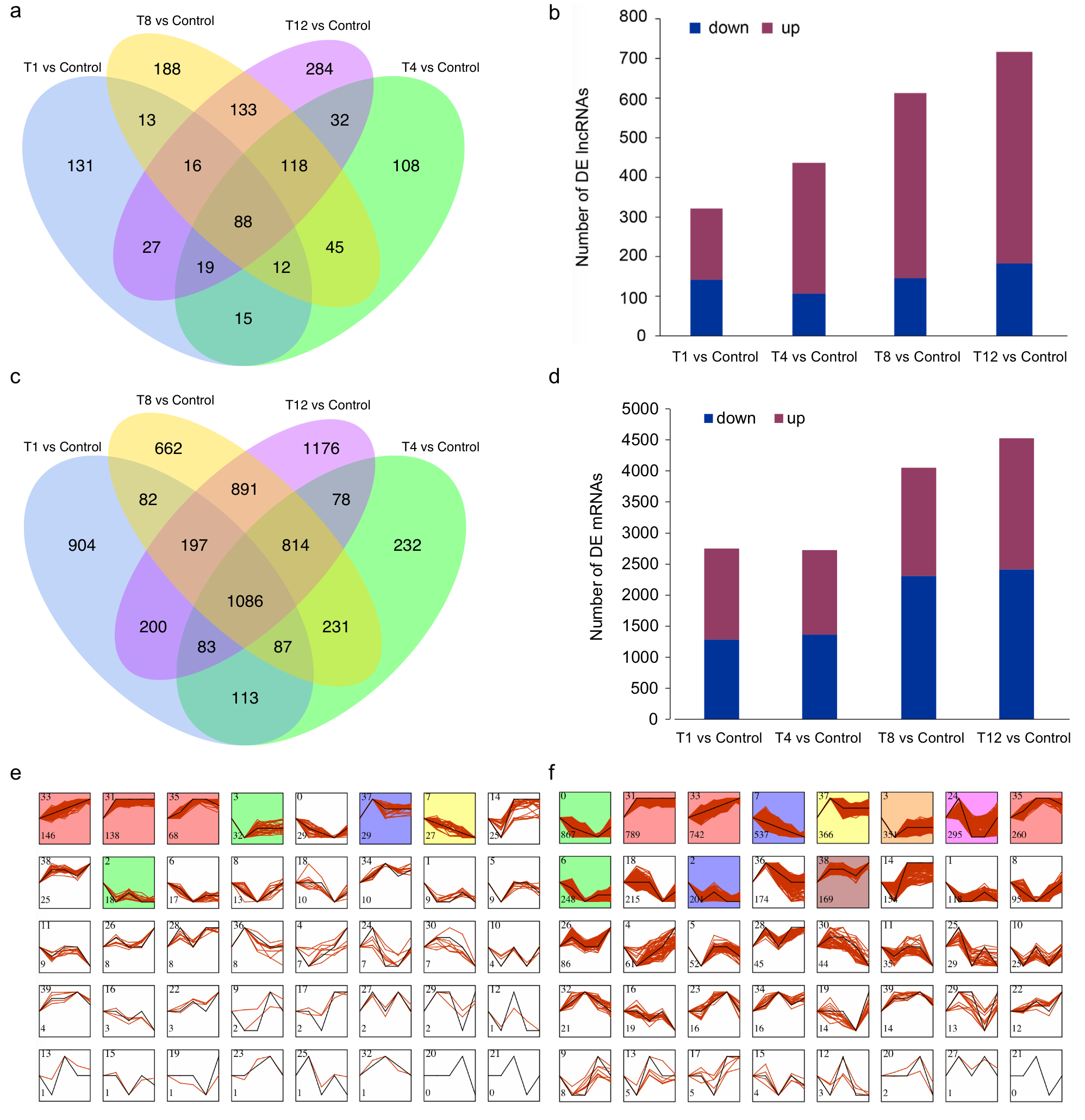


**Figure S6. Expression characteristics of differentially expressed (DE) lncRNAs and mRNAs in different comparison groups. (**a-b) The number of DE lncRNAs and their Venn diagrams between control and each treatment. (c-d) The number of DE mRNAs and their Venn diagrams between control and each treatment. Control sample represents plants before heating, T1, T4, T8, and T12 denotes plants that were subjected to 38°C for 1h, 4 h, 8h, and 12 h, respectively. (e-f) The expression patterns of DE lncRNAs and mRNAs identified by STEM clustering, respectively. A square represents a kind of expression mode, and a square with the same background color represents a similar expression mode. The black lines represent the model expression patterns, the red lines show the expression pattern of individual lncRNAs. The number in the upper-left corner represents the cluster ID, and the number in the lower-left corner represents the number of lncRNAs enriched in this cluster.

**Figure S7. The interaction relationship between DE lncRNAs and their *cis*-regulated DE mRNAs in Chinese cabbage.** (a) A combined DE lncRNA-mRNA interaction network of the four comparisons (T1, T4, T8, and T12, vs Control, respectively) constructed by Gephi software. Nodes represent lncRNAs (green) and mRNAs (red), respectively. Edges represent interaction between lncRNAs and mRNAs. Node size corresponds to the number of interacted mRNAs or lncRNAs. Edge color denotes the number of comparisons where different lncRNA-mRNA connections present. (b) Venn diagram shows the number of common and specific matched lncRNA-mRNA pairs in different comparisons. Black and grey dots represent the lncRNA-mRNA pair is present and absent in this comparison, respectively. (c) The number of adjacent mRNAs of lncRNAs. (d) The number of adjacent lncRNAs of mRNAs.

**Figure S8. Heatmap showing the expression patterns of lncRNAs and their *trans*-regulated *HSPs* or *HSP*-related genes.**


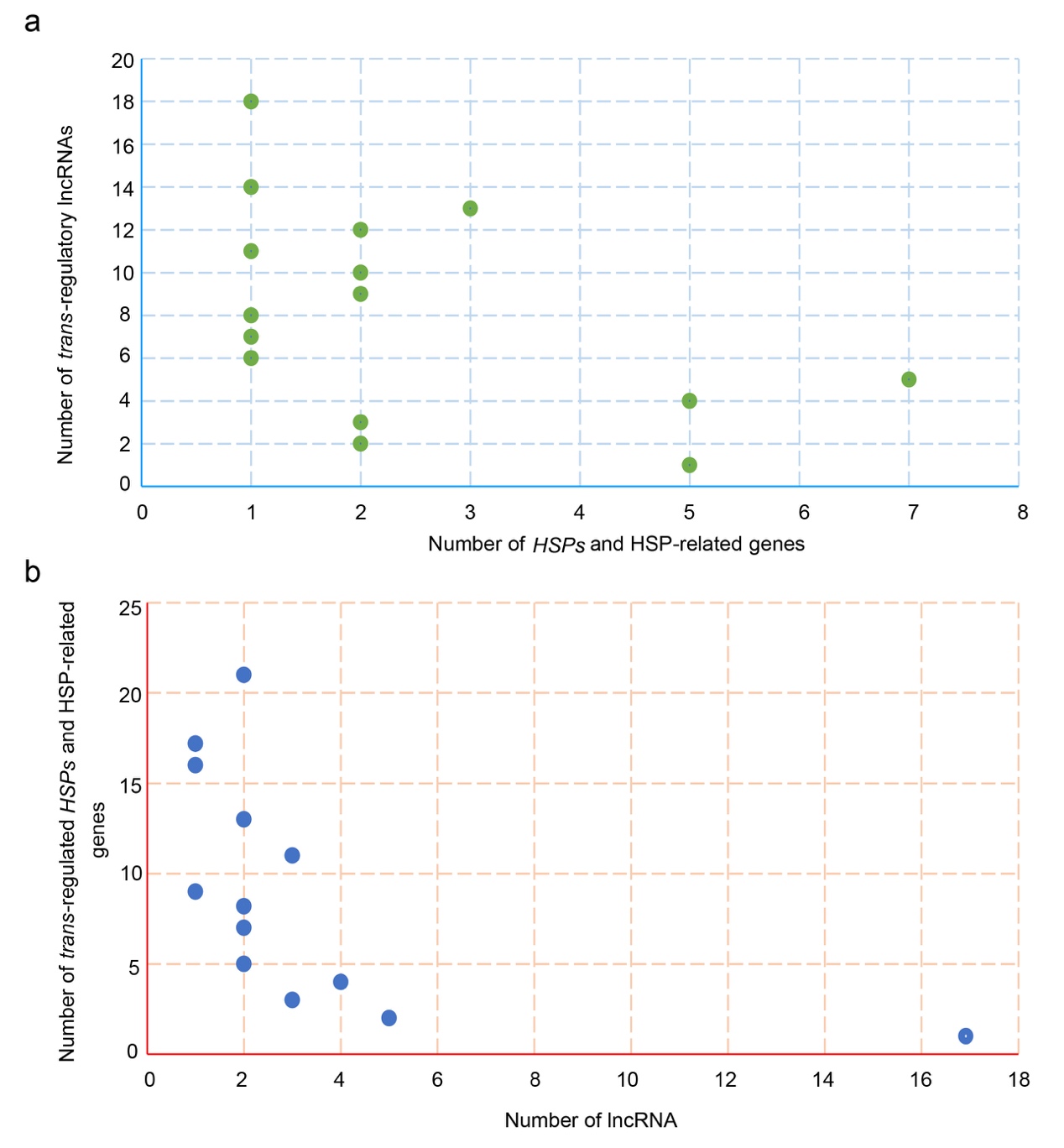


**Figure S9. The interaction relationship of DE lncRNAs with *HSPs* and *HSP*-related genes.** (a) The number of DE lncRNAs that have potential *trans*-regulatory effects on each *HSP* or HSP-related gene. (b) The number of *HSPs* and HSP-related genes *trans*-regulated by each DE lncRNAs.

**Figure S10. The expression patterns of protein ubiquitination associated genes enriched in 'protein processing in endoplasmic reticulum' pathway, and the regulatory network with their co-expressed lncRNAs.** (a) Heatmap showing the expression patterns of protein ubiquitination associated genes. (b) The interaction network between lncRNAs and protein ubiquitination associated genes. Nodes represent lncRNAs (green) and mRNAs (red). Node size corresponds to the number of interacted mRNAs or lncRNAs.

**Figure S11. The expression patterns of PYR/PYL family genes in 'plant hormone signal transduction ' pathway and the number of DE lncRNAs corresponding to each gene.** (a) Heatmap showing the expression patterns of PYR/PYL family genes. (b) The number of DE lncRNAs that have potential *trans*-regulatory effects on each PYR/PYL family gene.
